# Pediatricians at the forefront of child mental health? A Delphi method exploration

**DOI:** 10.1186/s13584-024-00661-5

**Published:** 2024-12-12

**Authors:** Shulamit Pinchover, Rony Berger Raanan, Hava Gadassi, Amit Shalev, Dasi Dahari, Tony Gutentag, Mary Rudolf

**Affiliations:** 1 Goshen Center, Jerusalem, Israel; 2 Haddassa Medical Center, Jerusalem, Israel; 3 Tel Aviv University, Tel Aviv, Israel; 4 Bar Ilan University, Ramat Gan, Israel

**Keywords:** Mental health, Pediatricians, Delphi method, Children, Mental health training

## Abstract

**Background:**

Rising mental health challenges among children and adolescents are a global concern. However, a shortage of professionals, inadequate competence and lack of resources hamper necessary care, presenting a major challenge to health service provision. Community pediatricians, frequently the initial contact for mental health issues, are in a key position to improve access to care. The Delphi method was employed as an initial step toward redefining the role of pediatricians and the re-design of pediatric responses within Israeli primary mental health care.

**Method:**

Ninety-two experts, including pediatricians, psychiatrists, mental health and child-development professionals, and parents of children with emotional-behavioral challenges participated in a three-round Delphi study. A survey including 6 topics (37 items, 9 demographic questions) was distributed, probing the envisioned role of pediatricians in children’s mental health care.

**Results:**

There was strong endorsement between experts regarding pediatricians’ potential roles in risk identification, parental guidance, psycho-education, and health policy isuues such as referrals to mental health professionals, and the need for training in this domain. However, discord arose concerning changes in the service framework and pediatricians’ role in psychiatric drug prescription. The majority agreed on the necessity of close support from mental health experts, despite differing in the form it should take.

**Conclusions:**

This study underscores the critical need for mental health training among pediatricians and advocates for a structured, consensus-driven model to bolster early detection and initial treatment of mental health issues in children. The findings highlight the potential for leveraging this model to drive health policy changes and improve service delivery in pediatric mental health care, and might inform other health systems considering extending pediatricians’ roles. By equipping pediatricians with essential competencies, this approach can broaden mental health service delivery and reduce stigma. Aligning the model with expert consensus paves the way for impactful policy reform, enhancing pediatricians’ roles in mental health risk identification and intervention, and advancing child health services.

## Background

In recent years, there is a concerning increase in children and adolescents diagnosed with emotional-behavioral challenges [1]. Between 10 and 20% in both developed and developing countries face mental health challenges [2]. In Israel, some 5% of children and teenagers aged 12–18 are affected by depression, 8% anxiety, 3% eating disorders, and 10% deal with stress [1]. The COVID-19 pandemic and the 2023 Hamas-Israel war has further exacerbated emotional/behavioral disturbances among this age group [3, 4]. Alarmingly, the country already faces a pronounced deficit of mental health professionals and resources [5], leading to prolonged waiting times and limited accessibility to quality care, particularly for minority communities and those in remote locations [6]. Such delays can result in adverse outcomes, including obstructing early identification, deteriorating mental health, escalating family conflict, and, in extreme cases, fatalities [7]. Innovative solutions and health policy enhancements are urgently needed to address these challenges.

Pediatricians, given their close and long-term interactions with children and families, are in a unique position to identify, intervene, and manage mental health concerns at an early stage [8, 9]. Traditionally, the primary focus of pediatrics has been on physical health; however, the intertwined nature of physical and mental well-being calls for a more holistic approach. According to the American Academy of Pediatrics, the pediatric role encompasses early detection, screening, provision of counseling and brief interventions, referrals, medication management, advocacy, and parental guidance [10]. Indeed, in some Western countries, including the USA, Canada, Australia, and Western Europe, pediatricians often serve as the first line of response for children’s mental health issues [11]. This evolving landscape highlights the urgency of ensuring that all pediatricians are competent in providing comprehensive healthcare.

## Redesigning the role of pediatrics to include mental health

Various models define the role of community-based pediatricians in mental health and guide the development of training programs, interventions and health policy. These models aim to foster expertise sharing and interprofessional collaboration in caring for young people with mental health challenges. They typically offer basic training, professional consultation, and a mechanism for identifying, responding to, and where necessary, referring children for specialist care [12, 13]. Evidence suggests that even brief training can significantly impact pediatricians’ practices and attitudes towards mental health challenges and improved care [11, 14, 15]. However, these training models often face challenges in sustainability, scope, and depth. Implementation varies significantly due to regional disparities in infrastructure and funding [11]. Enhancing these programs requires long-term investment, improved integration of mental health into primary care, and a focus on continuous adaptation and learning.

These limitations are exacerbated by structural differences in healthcare models between Israel and other countries. While some systems position pediatricians within secondary care, pediatric care in Israel operates mainly at the primary level. Training in psychosocial issues is fragmented, and support systems or mechanisms to assist pediatricians in mental health management are limited [16]. Consequently, community-based pediatricians frequently lack sufficient tools for identification of mental health conditions and primary care of those issues [16]. Furthermore, the pediatric service in Israel includes several barriers to pediatricians’ roles in addressing children’s mental health. For example, community pediatric services often provide only brief 5–10 min appointments, which limits the time available to adequately address mental health concerns. Additionally, there is a lack of systematic support from mental health professionals, further hindering pediatricians from effectively engaging with and managing these issues [16]. The gap in structured support in Israel highlights the need for further study and policy development to adapt international best practices to the unique characteristics of the Israeli healthcare system.

## The present investigation

We utilized the Delphi consensus method to explore the potential role of community-based pediatricians in child and adolescent mental health and to initiate reshaping their responsibilities, with the intention of gaining key stakeholder consensus regarding change in health service provision. Prior to starting this study, we received endorsements from the Israeli Pediatric and Psychiatry Associations, reinforcing the need to address pediatricians’ roles in mental health. The Delphi technique was selected due to its effectiveness in systematically collating expert opinions and building consensus among diverse groups, including pediatricians, psychiatrists, mental health and child development professionals (MHP), and parents. The Delphi method is particularly well-suited for this study as it allows for the inclusion of a wide range of perspectives, ensuring a comprehensive exploration of the complex and evolving roles pediatricians may undertake. It is valuable in navigating the dynamics between these diverse groups, where differences in professional expertise and personal experiences could otherwise lead to disparities in perceived authority or influence. By ensuring a structured and anonymous process, the Delphi method mitigates potential power imbalances, allowing each group—whether psychiatrists, pediatricians, or parents—to contribute equally without feeling marginalized or undervalued. The iterative nature of the Delphi method helps refine and prioritize these insights, ensuring a well-rounded and widely accepted consensus on the expanded roles pediatricians might undertake. Using this rigorous approach, the research was steered to achieve two primary goals: (1) To establish consensus regarding the kind of expanded role pediatricians might undertake in children’s mental health services, and (2) To identify the training and supervisory requirements essential for supporting pediatricians in this transition. By highlighting the critical need for targeted training and ongoing support, we aimed to pave the way for pediatricians to become integral in the mental health care ecosystem and to set the scene for significant but non-costly change in health policy regarding child mental health provision.

## Method

### The Delphi method

The Delphi approach is a structured method for binding together experts’ and stakeholders’ opinions in an attempt to reach a consensus on complex issues. It involves multiple rounds, with feedback provided between the rounds, to allow experts to refine their views. In each round, responses are gathered individually, ensuring that each participant is not influenced by group dynamics [17]. In the current study, a three-round Delphi method was employed [17]. Between rounds, participants received detailed feedback including statistics of other users’ answers, allowing them to refine their responses based on the group’s overall input. The Delphi method has been successfully utilized in the past to inform mental health and pediatric training and service design [18, 19]. The study was conducted according to the Guidance on Conducting and Reporting Delphi Studies (CREDES) [20].

### Expert panel

We focused on four groups of experts to ensure all key stakeholders were represented, given the broad scope of the research: (1) *Community pediatricians.* (2) *Child and adolescent psychiatrists.* (3) MHP – including policy makers, psychologists, social workers, educators and researchers in the field of child wellbeing/mental health. Inclusion criteria for professionals were at least 5 years of experience in their field. (4) *Parents*–with lived experience of parenting a child with a problem. Inclusion criteria for parents were having a child treated by a mental health specialist for at least 6 months.

**Fig. 1:**
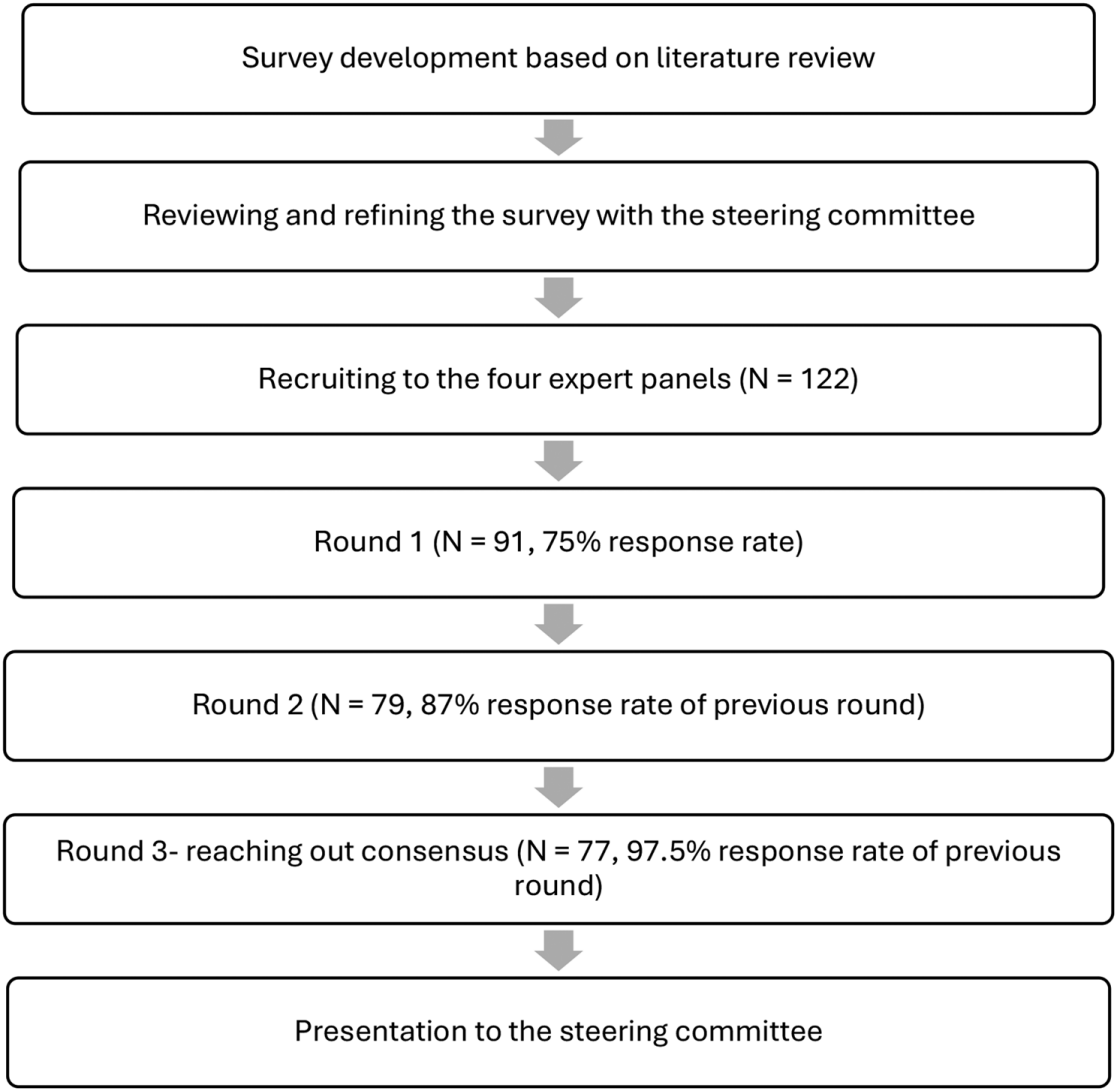
Delphi research procedure and response rates

To ensure the representativeness of the sample, we recruited participants based on a mapping of their expertise, influence, and experience. Significant figures in each professional field were identified through professional networks and relevant organizations, and then personally recruited to capture a wide range of perspectives. Parents were recruited through various support groups for children with mental health issues to provide a crucial perspective on lived experiences. Delphi study sample sizes vary, utilizing as few as ten and as many as hundreds of participants [17, 21]. To allow for anticipated attrition across rounds, we aimed to recruit 120 participants to ensure that at least 20 from each expert group completed the process. Of the 122 initially recruited, 91 (75%) completed the first round: 27 community pediatricians, 22 psychiatrists, 22 MHPs, and 20 parents; with little attrition in subsequent rounds. Their demographic characteristics are presented in Table 1, and the Delphi procedure and response rates in Fig. 1.

**Table 1:** Participant demographics (round 1, *N* = 91)

	All (*n* = 91)	Pediatricians (*n* = 27)	Psychiatrists (*n* = 22)	Parents (*n* = 20)	MHP* (*n* = 22)
Age (M +/- SD)	49.8 +/- 8.9	50.6 +/- 9.3	50.5+/- 8.4	49.0+/- 4.8	48.2 +/- 11.0
Gender (%, *n*)	Male(31.5%, 29) Female(68.5%, 62)	Male(46%, 13) Female(54%, 15)	Male(41%, 9) Female(59%, 13)	Male(5%, 1) Female(95%, 19)	Male(27%, 6) Female(73%, 16)
Sector (%, *n*)	Jews(90%, 82) Arabs(10%, 9)	Jews(86%, 24) Arabs(14%, 3)	Jews(86%, 19) Arabs(14%, 3)	Jews(95%, 19) Arabs(5%, 1)	Jews(91%, 20) Arabs(9%, 2)
Years in practice (M +/- SD)	20.8 +/- 10.8	19.8 +/- 13.1	22.3 +/- 7.9	---	21.1 +/- 11.03

*MHP: Mental health and child development professionals

### Construction of Delphi survey and analysis of items

A literature review was conducted between August to October 2022 for both ‘grey’ (policy statements and guidelines of mental health and pediatric associations, curricula of mental health training for pediatricians) and academic literature relating to the role of pediatricians in caring for children’s mental health around the world. Key papers were reviewed to identify potential areas for use in the Delphi survey. Survey items were then established by the research team. Items were presented to a steering committee composed of 12 leading pediatricians, psychologists, and psychiatrists, together with the resulting guidance. The committee provided feedback, leading to the finalization of the survey according to their comments. The final survey involved six main subjects, including 37 items, and 9 demographic questions. The areas covered were: (1) pediatricians’ roles in detection and prevention of children’s mental health (7 items), (2) pediatricians’ roles in providing an initial response to mental health concerns (8 items), (3) preferred service model for an initial pediatric response (5 items), (4) preferred training model (3 items), (5) training components (9 items), and (6) professional support and guidance (5 items). Options of agreement were presented on a five-point Likert scale for items in areas 1–2 and 4–6 (1 = *not relevant/needed at all*, 5 = *highly relevant/needed*). Two items in area 5 were open-ended, and one in area 3 was on a three-point scale (1 = *least favorite*, 3 = *most favorite*). Participants were encouraged to suggest any additional items or issues they felt were not already captured. To further enhance the clarity and effectiveness of the survey, the questionnaire was piloted with a small group of individuals to test the questions and review the provided materials. The inclusion of open-ended items in area 5 and a three-point scale in area 3 was a deliberate choice to capture nuanced feedback and preferences that might not be fully expressed through a standard Likert scale. These modifications were systematically applied to provide a more comprehensive understanding of the participants’ views and to better inform the development of training and support frameworks for pediatricians in mental health care.

#### Definition of consensus

An agreement of ≥ 70% between participants was taken to indicate consensus [21]. To determine levels of agreement or disagreement, we combined ‘highly relevant’ and ‘relevant’ to reflect agreement, whereas ‘not relevant/not needed at all’ and ‘not relevant’ were grouped to indicate disagreement. After each round, the survey items were categorized as follows: (1) *Endorsed*: a high rating from at least 70% of participants across groups, (2) *Requiring re-rating*: a rating from 50 to 69% of participants across groups, (3) *Rejected*: item did not meet criteria to be endorsed or re-rated (< 50%). Both consensus and non-consensus offer valuable insights and underscore the diverse perspectives on the topic, helping to identify areas for further exploration or clarification in the study.

Data collection was between March and May 2023. In Round 1, 16 items reached consensus. In Round 2, 20 items which had not reached consensus were re-presented, with 7 reaching consensus. In the third round, the consensus results were presented to all participants for approval. Participants were asked to confirm their agreement with the statement as it stood. No option to change previous responses was provided during this round. To ensure the validity and reliability of the final results, the project steering committee discussed the outcomes, further honing the consensus. Data analysis was conducted using both quantitative and qualitative methods, including percentage agreement across the panel and per group. The qualitative analysis involved thematic coding of open-ended responses. Percentage agreements were performed by Welphi after each round to identify areas of agreement and divergence.

## Results

### Role and service redesign

#### Pediatricians’ role in detection and prevention of children’s mental health problems

In round 1, three of the seven items reached consensus (> 70%). Two more items reached consensus in round 2 (Table 2). Items related to early detection received the highest consensus by all groups of experts. Screening for mental health issues was not considered by many to be part of the pediatricians’ role (Table 2).

**Table 2:** Pediatricians’ role in detection and prevention of mental health problems (% responding “very relevant/relevant”)

Round 1					
Item*	All (*N* = 91)	Pediatricians (*n* = 27)	Psychiatrists (*n* = 21)	Parents (*n* = 20)	MHP (*n* = 22)
**Identification of risk factors**	92%(84)	93%(25)	100%(21)	80%(16)	95%(21)
**Recognition of warning signs**	92%(84)	96%(26)	100%(21)	80%(16)	91%(20)
**Referral to information and tools**	78%(68, 3 missing)	74%(20)	80%(16, 1 missing)	84%(16, 1 missing)	71%(15, 1 missing)
Parental guidance about development and common disorders	65%(56, 5 missing)	56%(14, 2 missing)	67%(14)	70%(12, 3 missing)	68%(15)
Providing parental resources to reinforce resilience	62%(55, 3 missing)	72%(18, 2 missing)	71%(15)	47%(9, 1 missing)	55%(12)
Assessment of children presenting emotional-behavioral difficulties	58%(52, 4 missing)	52%(16)	57%(12)	65%(11, 3 missing)	62%(13, 1 missing)
Universal screening	34%(29, 6 missing)	37%(10)	30%(6, 1 missing)	25%(4, 4 missing)	43%(9, 1 missing)
**Round 2**					
Item*	**All** **(** ***N*** **= 79)**	**Pediatricians** **(** ***n*** **= 24)**	**Psychiatrists** **(** ***n*** **= 16)**	**Parents** **(** ***n*** **= 17)**	**MHP** **(** ***n*** **= 22)**
**Parental guidance about development and common disorders**	76%(61)	75%(18)	69%(11)	82%(14)	77%(17)
**Assessment of children presenting with emotional-behavioral difficulties**	74%(59)	75%(18)	62%(10)	82%(14)	73%(16)
Providing parents with resources to reinforce resilience	66%(53)	71%(17)	69%(11)	55%(11)	59%(13)
Universal screening	30%(24)	33%(8)	25%(4)	23%(4)	36% (8)

* Items in bold indicate consensus

#### Pediatricians’ role in providing an initial response to children’s mental health

In round 1, 4 of 8 items reached consensus across the panel, although differences were noticed between expert groups around two of them. In round 2, two additional items reached consensus (Table 3). In round 1, parents were hesitant about pediatricians providing an initial response. While *monitoring* and *management* of prescribed medication given by a psychiatrist/neurologist reached consensus in round 2, all groups agreed that *initiation* of prescribed medication should not be part of the pediatricians’ role.

**Table 3:** Pediatricians’ role in providing an initial response to children’s mental health (% responding “very relevant/relevant”)

Round 1					
Item*	All (*N* = 91)	Pediatricians (*n* = 27)	Psychiatrists (*n* = 21)	Parents (*n* = 20)	MHP (*n* = 22)
**Referral to a mental health professional**	93%(81, 3 missing)	100%(27)	100%(19, 2 missing)	79%(15, 1 missing)	91%(20)
**Referral to information and tools**	81%(71, 3 missing)	80%(22)	80%(16, 1 missing)	78%(14, 2 missing)	82%(18)
**Follow-up of the complaint and initiate intervention**	78%(67, 5 missing)	77%(20, 1 missing)	80%(16, 1 missing)	67%(12, 2 missig)	86%(18, 1 missing)
**Monitoring and management of prescribed medication given by a psychiatrist/neurologist**	72%(62, 5 missing)	59%(16)	75%(15, 1 missing)	67%(12, 2 missing)	90%(18, 2 missing)
Providing an ongoing response to common developmental issues	67%(58, 5 missing)	81%(22)	60%(12, 1 missing)	65%(11. 3 missing)	57%(12, 1 missing)
Case management/service coordination	64%(55, 5 missing)	59%(16)	75%(15, 1 missing)	62%(13, 2 missing)	50%(10, 1 missing)
Psychoeducation	59%(52, 3 missing)	52%(14)	70%(14, 1 missing)	53%(10, 1 missing)	62%(13, 1 missing)
Initiation of prescribed medication	39%(32, 9 missing)	27%(7, 1 missing)	21%(4, 2 missing)	43%(7, 4 missing)	65%(13, 2 missing)
**Round 2**					
Item*	**All** **(** ***N*** **= 79)**	**Pediatricians** **(** ***n*** **= 24)**	**Psychiatrists** **(** ***n*** **= 16)**	**Parents** **(** ***n*** **= 17)**	**(MHP** **(** ***n*** **= 22)**
**Monitoring and management of prescribed medication given by a psychiatrist/neurologist**	82%(64, 2 missing)	63%(15)	100%(16)	75%(12, 1 missing)	95%(20, 1 missing)
**Providing an ongoing response to common developmental issues**	79%(62, 2 missing)	83%(20)	71%(13)	81%(13, 1 missing)	71%(15, 1 missing)
Case management/service coordination	66%(52, 2 missing)	54%(13)	87%(14)	69%(11, 1 missing)	62%(13, 1 missing)
Psychoeducation	64%(50, 2 missing)	58%(14)	81%(13)	62%(10, 1 missing)	57%(12, 1 missing)
Initiation of prescribed medication	36%(28, 2 missing)	29%(7)	19%(3)	37%(6, 1 missing)	52%(11, 1 missing)

* Items in bold indicate consensus

#### Service model

Community pediatric services in Israel provide only a brief 5–10 min appointment per patient. In order to respond to mental health issues, the service model needs to be changed. In round 1, participants rated options from 1 (least favored) to 3 (most favored). Since they were able to rank more than one option, no clear consensus was reached: 49% (*n* = 44, 2 missing) favored a model where doctors could assign double appointments within clinic hours; 47% (42, 2 missing) preferred special “protected times” within working hours; 40% (*n* = 44, 3 missing)agreed that a designated district pediatrician would provide treatment for patients needing special support. In round 2, the question was modified to create more clarity, and participants were asked to indicate only their most favored option: 41% (*n* = 38) favored allowing doctors to assign a double appointment; 23% (*n* = 21) maintained a preference for “protected times” for longer appointments; and only 20% (*n* = 18) held on to the idea that a designated district pediatrician should provide the initial response to mental health issues. Although there was no absolute consensus, there was a preference for the model where doctors could assign double appointments within standard working hours for those requiring a more thorough response.

Participants were also asked in round 1 about an ideal but realistic duration for appointments needed to address children’s mental health issues. Average duration for first appointment was 36 min (*SD* = 13) and 24 min (*SD* = 11) for return appointments. Stakeholder groups did not differ significantly in responses. This question was not included in Round 2, as it was not deemed a consensus item, and no further clarification or differentiation among groups was needed.

### Training requirements

#### Training model

Globally, there are two principal models for mental health training for pediatricians: one focuses on generic competence in detecting and responding to children with emotional-behavioral challenges, the other provides more targeted training for specific common conditions. We asked for views regarding training in the Israeli context. In round 1, 66% (*n* = 60) preferred a generic approach to mental health, and in round 2, consensus was reached with 76% agreeing that training should be generic. No significant differences were found between groups.

#### Training components

In round 1, participants rated nine items related to skills and tools that should be included in future training (Table 4). Five of nine items reached consensus, with one additional item reaching consensus in the second round. Opinions diverged on items that did not reach a consensus. Pediatricians exhibited strong support for cognitive behavioral therapy tools and resources (83%), whereas psychiatrists (50%) and MHPs (38%) were less enthusiastic. These variances reflect the mental health landscape in Israel, which is heavily influenced by a robust psychoanalytic tradition. Parents tended to align with pediatricians, indicating an interest in practical tools for managing mental health concerns. All showed little agreement for screening instruments completed by children.

**Table 4:** Training components to be included in future training (% responding “*very relevant or relevant*”)

Round 1					
Item*	All (*N* = 91)	Pediatricians (*n* = 27)	Psychiatrists (*n* = 21)	Parents (*n* = 20)	MHP (*n* = 22)
**Clinical skills for initial assessment**	93%	93%(25)	100%(20, 1 missing)	82%(14, 3 missing)	95%(20, 1 missing)
**Clinical communication skills**	86%(74, 5 missing)	85%(23)	90%(18, 1 missing)	81%(14, 3 missing)	85%(18, 1 missing)
**Screening tools for pediatricians**	78%(68, 4 missing)	81%(22)	70%(14, 1 missing)	65%(11, 3 missing)	91%(18)
**Information sheets for parents about local resources**	79%(68, 5 missing)	85%(23)	85%(17, 1 missing)	75%(12, 4 missing)	68%(15)
Information sheets/digital resources for parental guidance	67%(58, 5 missing)	74%(20)	63%(12, 2 missing)	71%(12, 3 missing)	64%(14)
CBT tools and resources	57%(47, 9 missing)	70%(17, 3 missing)	47%(9, 1 missing)	62%(10, 4 missing)	45%(10)
Screening tools to be filled out by parents	53%(44, 8 missing)	54%(14, 1 missing)	53%(10, 2 missing)	33%(5, 5 missing)	68%(15)
Screening tools to be filled out by children	38%(42, 8 missing)	53%(14, 1 missing)	37%(7, 2 missing)	40%(6, 5 missing)	23%(5)
**Round 2**					
Item*	**All** **(** ***N*** **= 79)**	**Pediatricians** **(** ***n*** **= 24)**	**Psychiatrists** **(** ***n*** **= 16)**	**Parents** **(** ***n*** **= 17)**	**MHP** **(** ***n*** **= 22)**
**Information sheets/digital resources for guiding parents**	81%(62, 3 missing)	96%(22, 1 missing)	82%(13)	75%(12, 1 missing)	71%(15, 1 missing)
CBT tools and resources	61%(47, 3 missing)	83%(19, 1 missing)	50%(8)	69%(11, 1 missing)	38%(8, 1 missing)
Screening tools to be filled out by parents	61%(47, 3 missing)	61%(14, 1 missing)	62%(10)	43%(7, 1 missing)	76%(15, 1 missing)
Screening tools to be filled out by children	34%(36, 3 missing)	38%(9, 1 missing)	39%(6)	44%(7, 1 missing)	19%(3, 1 missing)

* Items in bold indicate consensus

Responses to open-ended questions (asked in round 1 only) on conditions demanding community pediatric competence included: anxiety (*n* = 33), depression (*n* = 33), ADHD (*n* = 17), emotional regulation or behavioral difficulties (*n* = 15), eating disorders (*n* = 14), and self-harm and suicide (*n* = 6). Specialists in mental health and child development indicated ADHD and emotional regulation difficulties more. In contrast, anxiety and depression were highlighted more by parents, psychiatrists, and pediatricians. Eating disorders were noted by both parents and pediatricians, whereas concerns regarding self-harm and suicide were exclusively raised by parents.

#### Professional support and guidance

Ongoing professional and expert guidance and peer support were viewed as crucial for long-term implementation and sustainability of an intervention. The survey sought views about the optimal format (Table 5). Two items initially reached consensus, with pediatricians favoring case studies and individual supervision, with 2 more items reaching consensus in Round 2.

**Table 5:** Professional support and guidance (% who responded “*very relevant/relevant*”)

Round 1					
Item*	All (*N* = 91)	Pediatricians (*n* = 27)	Psychiatrists (*n* = 21)	Parents (*n* = 20)	MHP (*n* = 22)
**Expert support hotline (by phone**,** email or WhatsApp)**	93%(79, 6 missing)	93%(25)	95%(18, 2 missing)	83%(15, 2 missing)	100%(20, 2 missing)
**Regular group case-study discussion led by a psychiatrist/psychologist.**	83%(71, 5 missing)	78%(21)	94%(15, 2 missing)	89%(16, 2 missing)	76%(16, 1 missing)
Individual supervision by child psychiatrist/psychologist	63%(54, 5 missing)	55%(15)	53%(10, 2 missing)	89%(16, 2 missing)	59%(12, 1 missing)
Professional consultation through online systems and medical files	62%(52, 7 missing)	70%(19)	63%(12, 2 missing)	61%(11, 2 missing)	47%(9, 3 missing)
Regular regional groups with psychiatrist/psychologist	62%(51, 9 missing)	57%(15, 1 missing)	57%(11, 2 missing)	78%(14, 4 missing)	50%(10, 2 missing)
**Round 2**					
Item*	**All** **(** ***N*** **= 79)**	**Pediatricians** **(** ***n*** **= 24)**	**Psychiatrists** **(** ***n*** **= 16)**	**Parents** **(** ***n*** **= 17)**	**MHP** **(** ***n*** **= 22)**
**Professional consultation through internal HMO service system**	75%(58, 4 missing)	91%(21, 1 missing)	69%(11)	68%(11, 1 missing)	67%(14, 1 missing)
**Individual supervision with child psychiatrist/psychologist**	71%(55, 3 missing)	65%(15, 1 missing)	62%(10)	88%(14, 1 missing)	71%(15, 1 missing)
Regular regional groups with psychiatrist/psychologist	69%(53, 3 missing)	65%(15, 1 missing)	75%(12)	87%(14, 1 missing)	57%(12, 1 missing)

* The items in bold indicate consensus

### Establishing the consensus

In the process of achieving consensus, a third round was conducted where experts were asked to review and approve the results. This was followed by a steering committee panel. Two primary subjects were discussed in particular. First, there was strong consensus on the importance of establishing a robust support system for pediatricians. Second, while most Delphi participants did not perceive prescribing psychiatric medication as part of the responsibility of pediatricians, there was active debate on this issue during the steering committee session. This highlighted the controversial nature of the subject, indicating the need for further consideration in later stages of program implementation. The results were approved by the committee.

## Discussion

The Delphi process underscored a wide consensus among all stakeholders concerning the need to redefine the role of pediatricians in identifying and delivering an initial response to children facing emotional challenges. Some notable differences between the responses of parents and professionals highlighted distinct perspectives within these groups. For instance, while pediatricians strongly supported providing an ongoing response to common developmental issues, psychiatrists and mental health professionals were less enthusiastic. There was also a notable difference in support for pediatricians monitoring and managing prescribed medication given by a psychiatrist or neurologist, with psychiatrists showing more support compared to pediatricians and mental health professionals. This difference may be related to the work overload psychiatrists face on one hand and the lack of confidence pediatricians have in managing psychiatric medications on the other. This variance reflects the influence of different professional training and practice philosophies [22]. Parents, on the other hand, showed alignment with pediatricians in their preference for practical tools for managing mental health concerns. Such differences underscore the need for tailored approaches in training and support systems to accommodate the diverse needs and viewpoints of all stakeholders involved in pediatric mental health care [23]. Stakeholders also started to set the boundaries for such involvement. While there was broad agreement regarding the pediatrician’s role in identifying early signs and risk factors, and referral to mental health specialists, agreement regarding additional responsibilities was less strong. The findings underscore the critical importance of specialized training and support for pediatricians, requiring ongoing professional development, enhanced collaboration with mental health professionals, and the implementation of ungoing proffesional suppurt system. We suggest a tiered model of care, where pediatricians provide initial mental health assessments and interventions but collaborate with mental health professionals for more complex cases, as one possible solution. This approach would prevent overwhelming pediatricians while ensuring appropriate level of care.

The controversial role of community-based pediatricians in prescribing or monitoring psychiatric medications poses significant discourse within the medical community. All participant groups were far from reaching consensus regarding the initiation of psychiatric medicine treatment. Previous studies have highlighted the controversial nature of this issue, with the management of psychiatric medications being seen as extending beyond pediatricians’ core expertise, potentially compromising the quality of care [22, 24]. It is argued that psychiatric drug prescription and monitoring should remain within the purview of psychiatrists, who possess the specialized training to navigate the complex landscape of psychiatric disorders and medications [22]. Supporters of a broader role for pediatricians point to the glaring gaps in psychiatric care, particularly in underserved regions, and argue that with appropriate training and resources, pediatricians can play a pivotal role in bridging these gaps [23]. Accordingly, some mental health training programs include teaching pediatricians to prescribe psychiatric drugs [11]. Our study emphasizes that at this time, pediatricians in Israel at least, should not take the place of psychiatrists in prescribing medicine, but can reduce the psychiatric workload by monitoring pre-prescribed medications.

While there was no full consensus on any specific service model, it is noteworthy that the two highest-rated models were similar in emphasizing the integration of pediatric mental health care into standard pediatric clinics. This similarity reinforces their suitability as preliminary guidance for policymakers, suggesting they may provide practical frameworks upon which further consensus can be built. We have therefore tempered these options, presenting these models as initial, rather than definitive, solutions. Integrating initial mental health care within primery pediatric settings may reduce the stigma linked to specialized psychiatry clinics, encouraging earlier and more frequent engagement with mental health services. Such an approach offers a more holistic, pragmatic, and comprehensive strategy to meet the extensive health needs of children, creating a supportive environment for both physical and mental health within a familiar healthcare setting. This approach also enables each HMO to examine and adapt the model that best suits its structure and specific needs, facilitating a tailored approach to service provision.

Our findings support a model where pediatric mental health care is integrated within standard pediatric clinics rather than establishing specialized clinics with implications for health policy. This favored approach may reflect an awareness of the stigma associated with child and adolescent psychiatry, a substantial obstacle that leads to undertreatment [25]. By providing initial mental health care within a regular clinic, the stigma may be reduced, promoting earlier and more frequent treatment engagement. It suggests an advocacy for seamless integration of mental health services into existing pediatric care frameworks, requiring change in health services provision and policy that potentially offers a more holistic, pragmatic and comprehensive method for meeting the extensive health needs of children [26].

To empower community-based pediatricians to effectively support children with emotional-behavioral challenges, it is critical to establish a robust support system [27]. The findings from the Delphi study emphasize a strong consensus among experts regarding this necessity, advocating for a personalized and responsive support infrastructure. In line with the recommendations by Constantino and Dilly [9], and within a comprehensive understanding of health—where mental health aspects cannot be separated from the overall physical health of children—this approach highlights the importance of integrated, collaborative frameworks to address the complex mental health needs of children. In the context of this study the onus of developing and sustaining this support system falls on Health Maintenance Organizations (HMO). These organizations are positioned to play a pivotal role in orchestrating a conducive environment wherein pediatricians can navigate the complexities of mental health care for children, alongside their physical health concerns. Moreover, by fostering a close-knit collaboration among pediatricians and mental health professionals a seamless continuum of care can be achieved. By transitioning towards a more integrated model of care, pediatricians can significantly contribute to a proactive and preventive mental health framework, promoting the holistic well-being of children in a manner that reflects contemporary understanding of health and disease. This redesign not only aligns with the broader global movement towards integrated healthcare but also sets a proactive stance in nurturing the mental health of the younger population, establishing a foundation for healthier communities in the long term [28].

Despite the encouraging findings, several potential implementation barriers to integrating mental health support into pediatric care should be noted. First, there may be logistical challenges, including the need to restructure clinic operations to allow for longer consultation times, which could create scheduling bottlenecks in clinics accustomed to time-limited appointments. Additionally, expanding pediatricians’ roles will require substantial investment in resources for both training and operational support. The Ministry of Health (MOH) and Health Maintenance Organizations (HMOs) may need to allocate budgets not only for initial and ongoing training but also for compensating pediatricians for the increased time spent with each patient. However, while investing in training pediatricians for mental health support presents upfront costs, it has the potential to reduce the long-term demand on specialized mental health services. Given the mental health crisis Israel is undergoing these days, the costs of healthcare are expected to rise exponentially. Creative ways to give mental health support, as fast as possible, are therefore an emergence. By equipping pediatricians to provide early intervention handle mild to moderate cases, this model should reduce referrals to specialists, making it cost-effective and ensuring that specialized resources are available for more severe cases [8]. Moreover, appropriate training will allow pediatricians to make more accurate referrals, which conserves time and resources for mental health specialists. In the macrolevel, the recommended policy changes would prove advantageous in direct and indirect costs on the health system.

### Research limitations and directions for future research

This Delphi study, based on a broad spectrum of key stakeholder insights, stands as a pivotal endeavor in synthesizing intricate relationships within mental healthcare services during a crucial period for young individuals and families. However, the sample did not entirely represent all groups and settings, marking this study as a starting point for more extensive research. While this composition reflects the typical demographics of those involved in pediatric mental health care, it may introduce a gender-related bias that could influence the perspectives represented in the consensus outcomes. Although this aligns with standard practices, a larger or more gender-diverse sample might have provided broader insights and potentially different consensus results. In addition, there was a slight reduction in participants during the second round, with some groups falling just below the 20-member threshold. However, since other studies using the Delphi method have produced reliable results with smaller sample sizes [17, 21], we expect that this minor decrease did not meaningfully impact the validity of the findings. Finally, due to the limited sample size for each expert group (*n* < 30), we focused on the overall consensus, and provided descriptive statistics per group, rather than analyzing significant differences between groups. Future research with larger sample sizes could enable more robust group comparisons.

Although this aligns with standard practices, a larger or more gender-diverse sample might have provided broader insights. Further studies should aim to include a more diverse range of settings and participants to enhance the applicability and relevance of the findings across different contexts. The methodology, while comprehensive, faced inherent limitations, such as only 75% of the initially recruited experts participated in the process, although target numbers were reached and those who participated in the first round stayed the course well. Moreover, while this study initiated a discourse on the pediatricians’ role and training needs, exploration of views through focus groups or qualitative interviews would add more dimensions to the findings Finally, this study begins to lay the foundation for change. Finally, this study begins to lay the foundation for change. Further research is needed to assess the feasibility and effectiveness of this model and to establish the necessary conditions for its larger scale implementation.

### Implications for policy and practice

The findings underscore the urgent need for innovative health policy solutions to address the strained mental health services for children and adolescents, a situation exacerbated by recent crises, including the COVID-19 pandemic and the ungoing Israeli-Hamas war. This study introduces a systemic solution for the early detection and initial response to children’s mental health issues, primarily through community-based pediatric services. Integrating comprehensive mental health training, developed in collaboration with mental health professionals and organizations, into the ongoing professional development of pediatricians has the potential to be a key strategy to influence health policy and service delivery. By equipping pediatricians with advanced mental health competencies, this approach can significantly expand mental health services to a broader population of children in need.

Given the global shortage of child and adolescent mental health professionals amidst the rising prevalence of mental health disorders, we recommend that policymakers ensure pediatric mental health training is incorporated as a standardized, nationally funded part of the medical curriculum for pediatricians. This curriculum should equip pediatricians to recognize early signs of mental health issues, offer brief interventions, and make referrals to specialists as needed. Additionally, facilitating the development of collaborative networks among pediatricians, mental health professionals, and community resources would create efficient referral pathways and foster integrated care models. These networks would enable regular consultation and ongoing support, bridging gaps between primary and specialist services. Structural adjustments within pediatric clinics are also essential; allowing extended consultation times for mental health cases, supported by policy incentives or targeted funding, would ensure that pediatricians have the time needed to address these concerns comprehensively.

To further support pediatricians, Health Maintenance Organizations (HMOs) should establish structured support systems, such as helplines, digital consultation platforms, and regular professional development workshops. Finally, it is essential to implement a monitoring and evaluation framework to assess the impact of these initiatives on child mental health outcomes and on healthcare efficiency. Pilot programs and feedback mechanisms could allow for iterative improvement, ensuring that policies are effective and responsive to emerging needs.

Underutilized in Israel, the Delphi methodology shows promise as a tool for accelerating policies that enhance child health care and well-being. This approach underscores the essential role of pediatricians in promoting early detection and treatment of mental health issues, directly benefiting children’s well-being.

## Conclusions

The study highlights a consensus among stakeholders on redefining the role of pediatricians in addressing children’s mental health challenges, underscoring the urgent need for health systems to adapt and integrate mental health resources into pediatric care. The findings have significant policy implications, suggesting a need for health systems and policymakers to integrate mental health training and resources into pediatric care frameworks, especially given the current global shortage of mental health professionals and the rising prevalence of mental health disorders among younger populations. The study underscores the necessity of tailored training and robust support systems for pediatricians to effectively bridge gaps in mental health care. By focusing on initial responses, monitoring pre-prescribed psychiatric medications, and integrating mental health care within pediatric settings, the study presents a pragmatic approach that could reduce stigma and improve accessibility to mental health services for children and adolescents. These recommendations lay the groundwork for scalable, evidence-based policy reform that prioritizes mental health within pediatric primary care, positioning Israel to meet growing mental health demands with a sustainable and locally relevant strategy.

## Data Availability

The datasets used and/or analysed during the current study are available from the corresponding author on reasonable request.

## Abbreviations

CREDES: Guidance on Conducting and Reporting Delphi Studies
MHP: Mental health and child development professionals
HMO: Health Maintenance Organizations
